# Landscape of 4D Cell Interaction in Hodgkin and Non-Hodgkin Lymphomas

**DOI:** 10.3390/cancers13205208

**Published:** 2021-10-17

**Authors:** Sylvia Hartmann, Sonja Scharf, Yvonne Steiner, Andreas G. Loth, Emmanuel Donnadieu, Nadine Flinner, Viola Poeschel, Stephanie Angel, Moritz Bewarder, Julia Bein, Uta Brunnberg, Alessandro Bozzato, Bernhard Schick, Stephan Stilgenbauer, Rainer M. Bohle, Lorenz Thurner, Martin-Leo Hansmann

**Affiliations:** 1 Dr. Senckenberg Institute of Pathology, Goethe University Frankfurt am Main, Theodor-Stern-Kai 7, 60590 Frankfurt am Main, Germany; Yvonne.Michel@kgu.de (Y.S.); nadine.flinner@kgu.de (N.F.); Julia.Bein@kgu.de (J.B.); 2 Frankfurt Institute of Advanced Studies, 60438 Frankfurt am Main, Germany; sonja.scharf@med.uni-frankfurt.de (S.S.); m.l.hansmann@em.uni-frankfurt.de (M.-L.H.); 3 Molecular Bioinformatics, Institute of Computer Science, Goethe University Frankfurt am Main, Robert-Mayer-Straße 11-15, 60325 Frankfurt am Main, Germany; 4 Department of Otolaryngology, Head and Neck Surgery, University Hospital Frankfurt, 60590 Frankfurt am Main, Germany; AndreasGerman.Loth@kgu.de; 5 Institut Cochin, INSERM U1016/CNRS UMR 8104, Université de Paris, 75014 Paris, France; emmanuel.donnadieu@inserm.fr; 6 Internal Medicine I, Saarland University Medical School, 66421 Homburg, Germany; Viola.Poeschel@uks.eu (V.P.); stephanie.angel@uks.eu (S.A.); Moritz.bewarder@uks.eu (M.B.); stephan.stilgenbauer@uks.eu (S.S.); lorenz.thurner@uks.eu (L.T.); 7 Department of Internal Medicine 2, Goethe University Hospital, 60590 Frankfurt am Main, Germany; uta.brunnberg@kgu.de; 8 Department of Otorhinolaryngology, Head and Neck Surgery, Saarland University Medical Center, 66421 Homburg, Germany; alessandro.bozzato@uks.eu (A.B.); bernhard.schick@uks.eu (B.S.); 9 Comprehensive Cancer Center Ulm (CCCU), University Hospital Ulm, 89070 Ulm, Germany; 10 Institute of Pathology, Saarland University Medical School, 66421 Homburg, Germany; Rainer.Bohle@uks.eu; 11 José Carreras Center for Immuno- and Gene Therapy, Saarland University Medical School, 66421 Homburg, Germany; 12 Institute of General Pharmacology and Toxicology, Goethe University Frankfurt am Main, Theodor-Stern-Kai 7, 60590 Frankfurt am Main, Germany

**Keywords:** migration, cell motility, cell contacts, Hodgkin lymphoma, non-Hodgkin lymphoma

## Abstract

**Simple Summary:**

Little is known about the motility and interaction of primary human lymphoma cells in lymph nodes. The aim of this study therefore was to analyze for the first time if there are differences in motility and interaction with bystander cells between different lymphoma types and normal lymph nodes. We observed systematic differences between B cells and PD1-positive T cells. Furthermore, most cases of Hodgkin lymphomas had fast moving PD1-positive T cells, whereas there was little movement in other lymphoma types. Some lymphomas, particularly Hodgkin lymphomas, presented enhanced cell contacts between neoplastic and reactive cells, suggesting a dependency of lymphoma growth on cellular interaction.

**Abstract:**

Profound knowledge exists about the clinical, morphologic, genomic, and transcriptomic characteristics of most lymphoma entities. However, information is currently lacking on the dynamic behavior of malignant lymphomas. This pilot study aimed to gain insight into the motility of malignant lymphomas and bystander cells in 20 human lymph nodes. Generally, B cells were faster under reactive conditions compared with B cells in malignant lymphomas. In contrast, PD1-positive T cells did not show systematic differences in velocity between reactive and neoplastic conditions in general. However, lymphomas could be divided into two groups: one with fast PD1-positive T cells (e.g., Hodgkin lymphoma and mantle cell lymphoma; means 8.4 and 7.8 µm/min) and another with slower PD1-positive T cells (e.g., mediastinal grey zone lymphoma; mean 3.5 µm/min). Although the number of contacts between lymphoma cells and PD1-positive T cells was similar in different lymphoma types, important differences were observed in the duration of these contacts. Among the lymphomas with fast PD1-positive T cells, contacts were particularly short in mantle cell lymphoma (mean 54 s), whereas nodular lymphocyte-predominant Hodgkin lymphoma presented prolonged contact times (mean 6.1 min). Short contact times in mantle cell lymphoma were associated with the largest spatial displacement of PD1-positive cells (mean 12.3 µm). Although PD1-positive T cells in nodular lymphocyte-predominant Hodgkin lymphoma were fast, they remained in close contact with the lymphoma cells, in line with a dynamic immunological synapse. This pilot study shows for the first time systematic differences in the dynamic behavior of lymphoma and bystander cells between different lymphoma types.

## 1. Introduction

Malignant lymphomas are classified into specific entities according to the 2017 updated 4th edition of the “WHO classification of tumours of haematopoietic and lymphoid tissues” [1]. A broad knowledge exists about the clinical, morphological, and genetic characteristics of most entities [2,3,4,5,6]. However, little is known of the dynamics of lymphoma cells. This issue is of importance, since motility is an inherent property of lymphoid cells. Lymphoid cells can easily migrate in an amoeboid manner without reorganization of the extracellular matrix [7,8], whereas cancer cells of solid tumors frequently employ a mesenchymal migration type [9]. Therefore, the active motility of lymphoma cells is important because it allows easy spread to distant sites of the human body, yielding different disease stages at diagnosis. For the moment, our knowledge of lymphoma cell migration comes essentially from in vitro or in vivo experiments performed in mouse models [10]. The migration properties of human lymphomas have so far been characterized in vitro using established cell lines [11,12,13,14]. Linke et al. identified *WNT5A* as an important factor for the amoeboid cell migration of human lymphoma cells [15]. However, the function of lymphoma cells in their natural environment (e.g., lymph nodes) remains ill-defined. The microenvironment of lymphomas, which has been deeply characterized [5,16,17], differs in microenvironment-rich types, such as all variants of Hodgkin lymphoma, and others that are rich in tumor cells and poor in bystander cells, such as most mature B-cell lymphomas. Lymphoma cells interact at different levels with their bystander cells. In diffuse large B-cell lymphoma, where the microenvironment is frequently scarce, a stromal signature of the microenvironment has been correlated with a worse prognosis [18]. Thus, even in entities with a low abundance of microenvironmental cells, their role can be relevant. Malignant lymphoma cells interact with microenvironmental cells in various manners [19]. Immunological synapses are formed between B cells and follicular T helper (T_FH_) cells in reactive lymph nodes and particularly in lymphomas with germinal center derivation, such as follicular lymphoma or nodular lymphocyte-predominant Hodgkin lymphoma [20,21]. The interaction between B cells and T_FH_ cells may provide important survival signals to the malignant clone [22,23]. However, little is yet known about the impact of these immunological synapses on lymphoma growth and motility or the time frames in which these cellular interactions persist.

This pilot study therefore aimed to elucidate differences in the motility between lymphoma and bystander cells to learn about their interactions, as well as differences between lymphomas such as Hodgkin lymphoma and mature B-cell lymphomas. For the first time, to our knowledge, we characterize here the motility and dynamic interaction with bystander cells in thick slices of primary human lymphomas.

## 2. Materials and Methods

### 2.1. Embedding and Live Cell Imaging

The tissue of 20 primary lymph nodes was investigated. Informed consent of all patients was obtained in accordance with the Declaration of Helsinki. The local ethics committees (University Hospital Frankfurt No. 387/16 and Landesärztekammer des Saarlandes Ha76/19) approved this study.

After surgical removal of the suspicious lymph node and embedding of representative tissue for the routine diagnostic process, a 2-mm piece of the remaining tissue was embedded into 5% low-gelling-temperature agarose (Type VII-A; Merck, Darmstadt, Germany) prepared in PBS as previously described [24,25]. Slices of 350 µm were cut with a vibratome (VT1000S; Leica Biosystems, Nussloch, Germany) in a bath of ice-cold PBS. The slices were then transferred to 0.4-mm organotypic culture inserts (Millicell; Millipore, Burlington, MA, USA) in 35-mm Petri dishes containing 1.1 mL of RPMI 1640 without phenol red. Slices were then kept on ice until antibody staining and imaging was performed, usually less than 3 hrs. Live vibratome sections were stained for 15 min at 37 °C with the following antibodies: Alexa Fluor 647–anti-human PD-1 (clone EH12.2H7; BioLegend, San Diego, CA, USA), Alexa Fluor 647–anti-human CD30 (clone CD30/412, Novus Biologicals, Centennial, CO, USA), Alexa Fluor 488–anti-human PD-1 (clone EH12.2H7; BioLegend), Alexa Fluor 647–anti-human CD3 (clone UCHT1; BD Biosciences), Brilliant Violet 421–anti-human CD3 (clone UCHT1; BioLegend), Alexa Fluor 488–anti-human CD19 (clone HIB 19; BioLegend), Pacific Blue–anti-human CD20 (clone 2H7; BioLegend), Alexa Fluor 555-anti-human CD20 (polyclonal rabbit antibody; Bioss Antibodies, Woburn, MA, USA), and Alexa Fluor 555-anti-human CD19 (polyclonal rabbit antibody; Bioss Antibodies).

All antibodies were diluted in RPMI 1640 without phenol red and used at a concentration of 10 µg/mL. To concentrate the antibodies on the tissue, a stainless steel ring was placed on the agarose surrounding the slice. The tissue slices were imaged with a Leica SP8 confocal microscope (Leica Microsystems) equipped with a 37 °C thermostatic chamber. The temperature was constant, and oxygen was controlled by self-written appropriate software. For dynamic imaging, the tissue slices were secured with a stainless steel slice anchor (Warner Instruments) and perfused at a rate of 0.8 mL/min with a solution of RPMI 1640 without phenol red, bubbled with 95% O_2_ and 5% CO_2_. For four-dimensional analysis of cell migration, stacks of 10–12 sections (z step = 5 µm) were acquired every 10–20 s for 20 min at depths up to 80 µm.

### 2.2. Data Analysis

Cellular motility parameters were calculated after three-dimensional reconstruction of the sequential z series using Imaris software. Automated tracking of the respective cells was done by the Spots tool in Imaris. The “Estimated Diameter” settings were 7 µm for CD3- and PD1-positive cells, 8 µm for CD19- and CD20-positive cells, and 10 µm for CD30-positive cells. The filter threshold was adjusted by visual inspection. Velocities were calculated according to the tracking tool “Autoregressive Motion.” The settings were “MaxDistance” not less than cell size, “MaxGapSize” 3, and “Fill Gap Enable” true. Calculation of cell–cell contacts assessed the number of cells closer than a defined distance to the center of another cell. According to the surface staining, first, the diameter of all PD1-, CD20-, and CD30-positive cells was calculated in three dimensions. The mean diameters were 7 µm for PD1- and CD3-positive cells, 8 µm for CD20- and CD19-positive cells, and 10 µm for CD30-positive cells. A cell–cell contact was assumed to be present when the distance between two cells was less than the sum of their radii (distance between CD20- and PD1-positive cells or CD19- and CD3-positive cells: 7.5 µm from center to center; distance between CD30- and PD1-positive cells: 8.5 µm). For statistical analyses, Gaussian distribution was tested using the Kolmogorov–Smirnov test. For two-group comparisons, either an unpaired *t*-test or the Mann–Whitney test was performed. Comparison of more than two groups used a one-way analysis of variance (ANOVA) with Bonferroni’s post-test for multiple comparisons or a Kruskal–Wallis test with Dunn’s post-test for multiple comparisons.

## 3. Results

### 3.1. Cases

The lymph nodes analyzed represented five cases of lymphadenitis, comprising two cases of infectious mononucleosis, two cases of non-specific lymphadenitis, and one case of Kikuchi-Fujimoto disease. Additionally included were two cases of diffuse large B-cell lymphoma (DLBCL), three cases of follicular lymphoma (FL) grade 1–2, one case of mediastinal grey zone lymphoma (MGZL) and the corresponding relapse in the same patient, three cases of classical Hodgkin lymphoma (cHL), two cases of nodular lymphocyte-predominant Hodgkin lymphoma (NLPHL), two mantle cell lymphomas (MCL), and one angioimmunoblastic T-cell lymphoma (AITL).

All fresh, non-fixed, tumors were cut in thick slices (350 µm) and then were stained with a fixed combination of fluorescent antibodies directed against CD20 (normal and malignant B cells) and PD1 (T_FH_ and activated T cells). In addition, variable antibody combinations including CD30 (Hodgkin and Reed–Sternberg cells), CD19 (normal and malignant B cells), and CD3 (all T cells) were performed since not all malignant cells were positive for CD20. Confocal dynamic imaging experiments were then performed to track stained cells during a 20 min recording within an intact lymph node environment. A total of 80,400 moving live cells were analyzed for their velocity and cell–cell contacts, as described in Table 1.

### 3.2. PD1-Positive T Cells Move Faster Than CD20-Positive B Cells and CD30-Positive Cells

**Figure 1:**
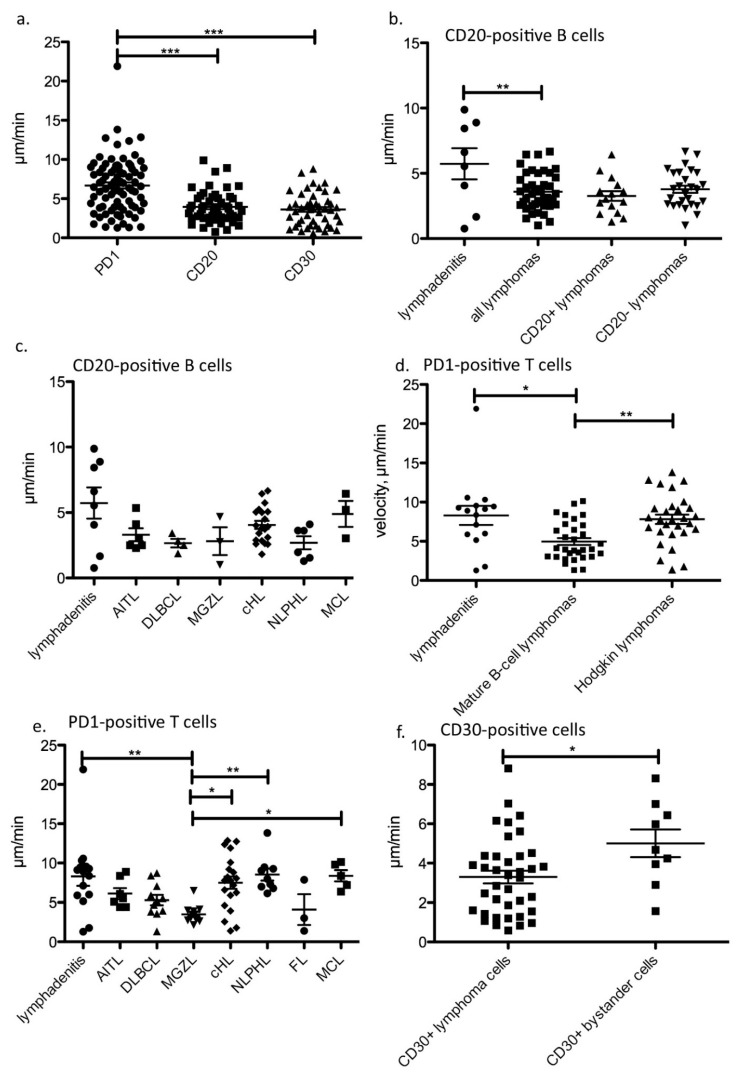
Velocity of different cell types under reactive and neoplastic conditions. (**a**). Velocity of PD1-positive T cells, CD20-positive B cells and CD30-positive cells in all cases studied (*** *p* < 0.001, one-way-ANOVA with Bonferroni’s post-test for multiple comparisons). Each dot represents the mean from one movie. (**b**). Velocity of CD20-positive B cells in lymphadenitis and malignant lymphomas. The velocity of bulk CD20-positive B cells in CD20+ lymphomas largely represents the velocity of tumor cells, whereas the velocity in CD20- lymphomas corresponds to reactive bystander B cells. (** *p* = 0.0042, unpaired *t*-test.) Each dot represents the mean from one movie. (**c**). Velocity of CD20-positive B cells listed according to different lymphoma entities. No significant differences were observed. Each dot represents the mean from one movie. Lymphadenitis: 4 cases, AITL, DLBCL and MCL: one case, MGZL and NLPHL: two cases, cHL: three cases. (**d**). Velocity of PD1-positive T cells grouped according to diagnosis of lymphadenitis, mature B-cell lymphomas and Hodgkin lymphomas. Each dot represents the mean from one movie. * *p* < 0.05, ** *p* < 0.01, Kruskal–Wallis test with Dunn’s post-test for multiple comparisons. (**e**). Velocity of PD1-positive T cells according to lymphoma entities. ** *p* < 0.01, *** *p* < 0.001, Kruskal–Wallis-Test with Dunn´s post-test for multiple comparisons. Each dot represents the mean from one movie. Lymphadenitis: 5 cases, AITL and MCL: one case, MGZL, DLBCL, FL and NLPHL: two cases, cHL: three cases. (**f**). Velocity of CD30-positive lymphoma cells compared with CD30-positive reactive bystander cells in CD30-negative lymphomas and lymphadenitis (* *p* < 0.05, unpaired *t*-test). Each dot represents the mean from one movie.

In most lymph nodes investigated, the motility of CD20-positive B cells was recorded simultaneously with that of PD1-positive T_FH_ cells. Therefore, the velocity of B and T cells could be ideally compared in the same lymph node and the same movies. On average, PD1-positive T cells, representing both T_FH_ as well as activated T cells, were significantly faster than bulk B cells and CD30-positive cells (mean velocities 6.68 µm/min vs. 3.94 µm/min and 3.62 µm/min, *p* < 0.0001, one-way ANOVA with Bonferroni’s multiple comparison test; Figure 1a).

### 3.3. B Cells Are Faster in Reactive Than in Neoplastic Conditions

When comparing the velocity of CD20-positive B cells, B cells in the lymphadenitis specimens were generally faster than bulk B cells in malignant lymphomas (mean 5.73 vs. 3.60 µm/min, *p* = 0.0042, unpaired *t*-test; Figure 1b). Interestingly, among the lymphomas studied, there was no difference whether the B cells belonged to the neoplastic clone (CD20-positive lymphomas) or were normal B cells in a remodeled lymph node infiltrated by CD20-negative lymphomas (e.g., in AITL or Hodgkin lymphomas, Figure 1b). Although no significant differences existed in the velocity of B cells among the different types of lymphomas, B cells in MCL were the fastest B cells within a lymphoma (mean 4.90 µm/min), which is faster than B cells in DLBCL (mean 2.67 µm/min, Figure 1c).

### 3.4. The Fastest PD1-Positive T Cells Occur in NLPHL and MCL

**Figure 2:**
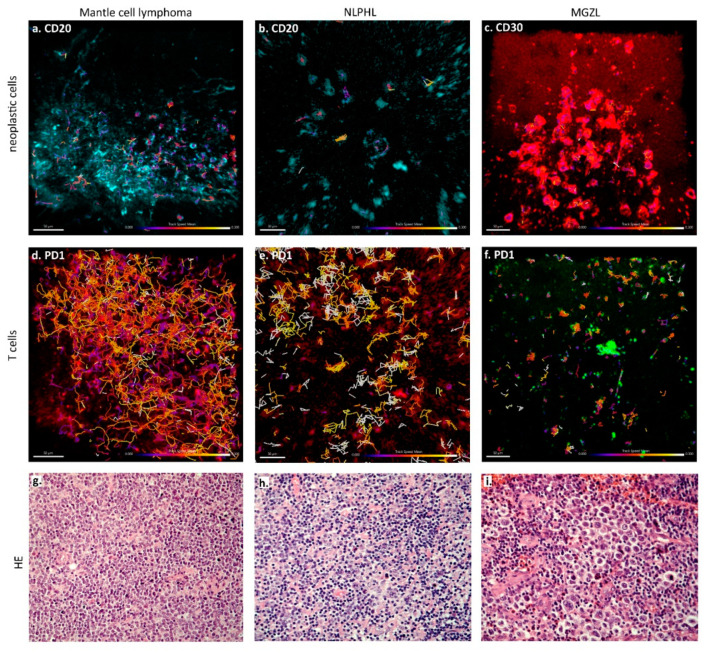
Cell tracks according to different lymphoma types superimposed on fluorescence images. (**a**–**c**) Tracks of the tumor cells of mantle cell lymphoma (**a**), NLPHL (**b**) and MGZL (**c**) in CD20- or CD30-immunostaining, as indicated. Color of tracks corresponds to the observed velocity as indicated in the scale. (**d**–**f**) Tracks of the bystander T cells of the same cases: mantle cell lymphoma (**d**), NLPHL (**e**) and MGZL (**f**) in PD1-staining. Color of tracks corresponds to the observed velocity as indicated in the scale. (**g**) HE staining (200x) of the mantle cell lymphoma from (**a**,**d**). (**h**) HE staining (200x) of the NLPHL from (**b**,**e**). (**i**) HE staining (200×) of the MGZL from (**c**,**f**).

In contrast to B cells in lymphadenitis and lymphoma specimens, no significant difference existed in the mean velocity of PD1-positive T cells between lymphadenitis and lymphomas in general (means of 8.31 µm/min vs. 6.35 µm/min). However, PD1-positive T cells were significantly faster both in Hodgkin lymphomas and lymphadenitis (mean velocities of 7.81 and 8.31 µm/min) when compared with mature B-cell lymphomas (mean velocity of 4.97 µm/min, *p* < 0.05, ** *p* < 0.01, *** *p* < 0.001, Kruskal–Wallis test with Dunn´s post-test for multiple comparisons, Figure 1d). PD1-positive T cells were the fastest in NLPHL (mean velocity 8.53 µm/min), MCL (mean velocity 8.37 µm/min), and lymphadenitis (mean velocity 8.31 µm/min), and were significantly faster than PD1-positive T cells in MGZL (mean velocity 3.49 µm/min, *p* < 0.01, Kruskal–Wallis test with Dunn’s post-test for multiple comparisons; Figure 1e and Figure 2, Suppl. Movies S1 and S2).

### 3.5. CD30-Positive Normal Cells Move Faster Than CD30-Positive Lymphoma Cells

CD30 immunostaining was performed in several cases of lymphadenitis as well as CD30-positive and -negative lymphomas. CD30 is expressed in the tumor cells of some lymphomas like cHL, MGZL and EBV-associated DLBCL. However, CD30-positive cells are also found under almost all reactive conditions and also occur as CD30-positive bystander cells, usually activated T cells, in CD30-negative lymphomas. We thus analyzed velocity separately in the different lymphoma types depending on CD30-positivity of the tumor cells, which was the case for all cHL and MGZL and one case of EBV-associated DLBCL. CD30-positive tumor cells showed a mean velocity of 3.29 µm/min. CD30-positive bystander cells were slightly faster, with a mean velocity of 5.00 µm/min observed in CD30-negative lymphomas and lymphadenitis (*p* = 0.01, Mann–Whitney test; Figure 1f). Interestingly, the CD30-positive tumor cells of the EBV-associated DLBCL were significantly faster (5.58 µm/min) than the CD30-positive tumor cells in MGZL (2.69 µm/min, *p* < 0.05, Kruskal–Wallis test with Dunn’s post-test for multiple comparisons; Supplementary Figure S1). The Hodgkin and Reed–Sternberg cells in cHL showed a comparable velocity (mean 3.31 µm/min) to MGZL, but this was not significantly different from DLBCL.

### 3.6. The Number of Contacts between B Cells and T Cells Is Similar in Reactive and Neoplastic Conditions

**Figure 3:**
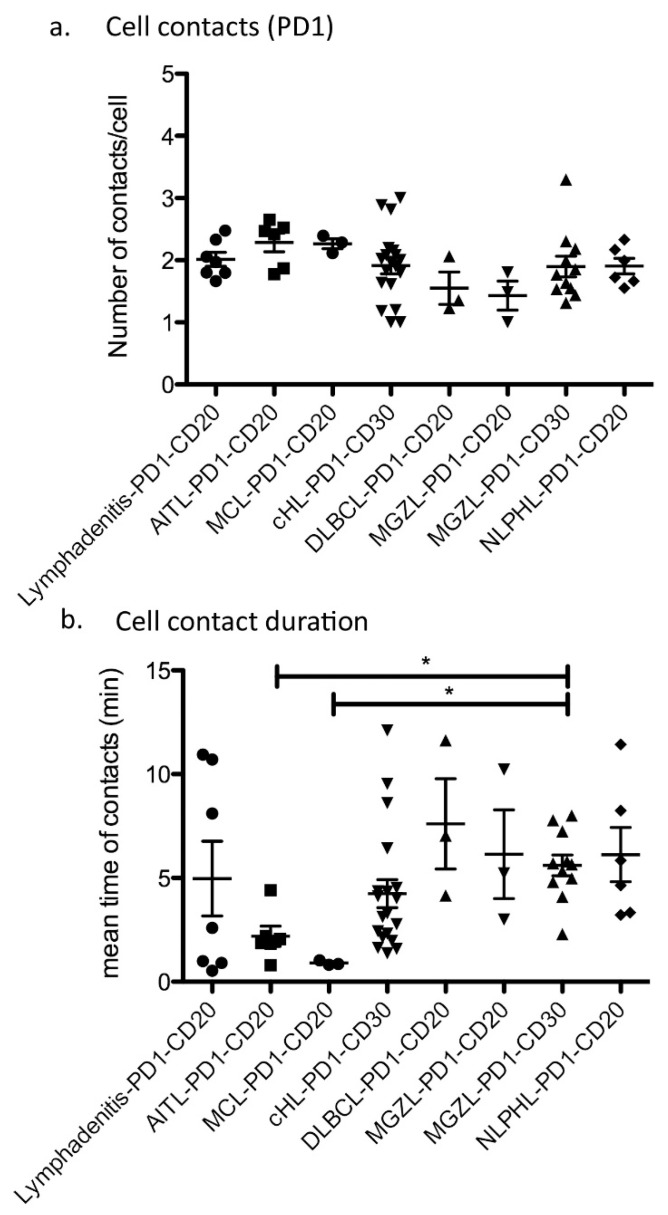
Cell–cell contacts between tumor and bystander cells. (**a**) Number of cell–cell contacts per movie between tumor cells (CD20 or CD30 as indicated) and PD1-positive T cells (for AITL PD1-positive tumor cells and CD20-positive B cells). Each dot represents the mean from one movie. Lymphadenitis: 4 cases, AITL, DLBCL, NLPHL and MCL: one case, MGZL: two cases, cHL: three cases. (**b**) Duration of cell–cell contacts between tumor (CD20 or CD30 as indicated) and PD1-positive T cells (for AITL PD1-positive tumor cells and CD20-positive B cells, * *p* < 0.05, Kruskal–Wallis test with Dunn’s post-test for multiple comparisons). Each dot represents the mean from one movie. Lymphadenitis: 4 cases, AITL, DLBCL, NLPHL and MCL: one case, MGZL: two cases, cHL: three cases.

We assessed not only the velocity of the cells, but also the number of contacts between different cell types, as well as the contact durations during the whole recording (20 min). Since we studied different lymphoma types, tumor cells presented variable immunophenotypes, with some lymphomas having CD20-positive lymphoma cells and others having CD30-positive lymphoma cells, which makes comparing tumor–bystander cell contacts with fixed antibody combinations difficult. To make the best possible comparison, we selected for B-cell lymphomas and Hodgkin lymphomas a tumor-specific antigen (either CD20 or CD30) and assessed the number of PD1-positive T cells that were closer than a defined distance to the center of the tumor cell. Since the tumor cells of AITL are PD1-positive T cells, we assessed the interaction of the tumor cells with CD20-positive normal B cells in this context. The number of cell–cell contacts between CD20-positive B cells and PD1-positive T cells was similar in lymphadenitis and the different lymphoma types (average 1.92 contacts per movie and per cell for CD20–PD1 interaction). The same was true for the number of CD30-positive–PD1-positive cell contacts (average 1.93 contacts per movie for CD30–PD1 interaction; Figure 3a). Since we had additionally acquired some movies with CD3 staining, which includes broader subsets of T cells than those stained by anti-PD1 antibodies, we also analyzed these for the number of contacts formed. Specifically, we studied contacts between CD3-positive T cells and CD19-positive reactive B cells in lymphadenitis and neoplastic B cells in DLBCL as well as CD30-positive lymphoma cells in MGZL, cHL, CD30-positive DLBCL. However, the number of contacts per movie and per cell that were observed for CD3-positive T cells was comparable to that observed for PD1-positive T cells (mean of 2.14 contacts; Supplementary Figure S2), therefore likely representing the number of cells encountered by a T cell at random walk.

### 3.7. Different Lymphoma Entities Differ in the Duration and Manner of Cell–Cell Contacts

**Figure 4:**
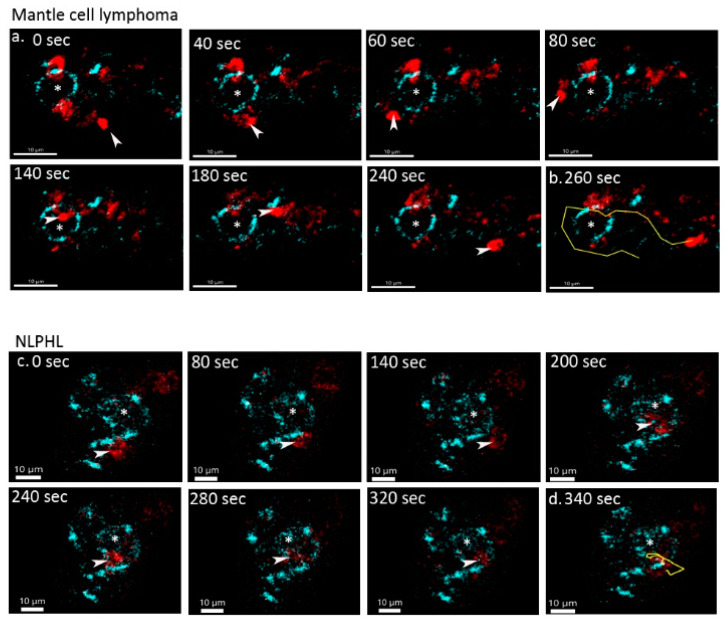
Variable duration of cell–cell contacts in different lymphomas. (**a**) Time lapse images of a PD1-positive T cell (red, marked by arrow head) undergoing a short contact with a CD20-positive lymphoma cell (blue, marked by *) in mantle cell lymphoma. (**b**) Track (yellow) of the PD1-positive T cell from the mantle cell lymphoma from a. (**c**) Time lapse images of a PD1-positive T cell (red, marked by arrow head) undergoing a long dynamic contact with a CD20-positive lymphoma (LP) cell (blue, marked by *) of NLPHL. (**d**) Track (yellow) of the PD1-positive T cell from the NLPHL from c.

**Figure 5:**
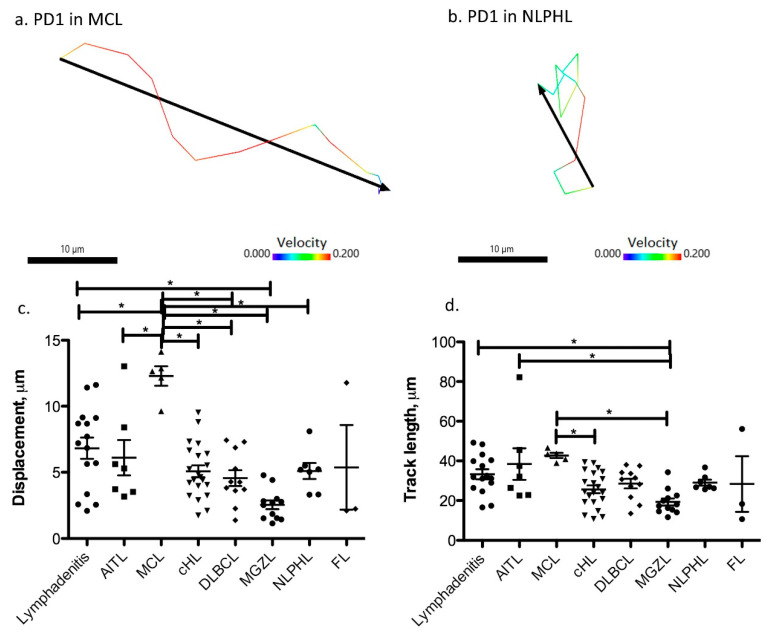
Lymphomas differ in track length and displacement of their PD1-positive T cells. (**a**) Track length (color code according to velocity in µm/s) and absolute displacement (black) of an example of a PD1-positive T cell in mantle cell lymphoma. (**b**) Track length (color code according to velocity in µm/s) and absolute displacement (black) of an example of a PD1-positive T cell in NLPHL. (**c**) Absolute displacement of PD1-positive T cells in different lymphomas (* *p* < 0.05, one-way ANOVA with Bonferroni’s post-test for multiple comparisons). Each dot represents the mean from one movie. Lymphadenitis: 5 cases, AITL, NLPHL and MCL: one case, MGZL, DLBCL and FL: two cases, cHL: three cases. (**d**) Accumulated track length of PD1-positive T cells in different lymphomas (* *p* < 0.05, one-way ANOVA with Bonferroni´s post-test for multiple comparisons). Each dot represents the mean from one movie. Lymphadenitis: 5 cases, AITL, NLPHL and MCL: one case, MGZL, DLBCL and FL: two cases, cHL: three cases.

Analyzing the contacts between different types of reactive and neoplastic B and T cells, we found significant differences in the duration of cell–cell contacts. In lymphadenitis, both short and long-lasting contacts were observed between PD1-positive T cells and CD20-positive B cells (Suppl. Movies 3 and 4). AITL and MCL were the lymphomas with the shortest contact times between PD1-positive and CD20-positive cells (means of 2.19 and 0.91 min), whereas NLPHL, MGZL, and one case of DLBCL (GCB-type) showed enhanced contact times between lymphoma cells and PD1-positive T cells (means of 6.12, 5.61, and 7.61 min, respectively; Figure 3b and Figure 4). In cHL, a large spectrum of cell contact durations ranging from short cellular contacts to long lasting interactions between PD1-positive T cells and Hodgkin-Reed-Sternberg cells occurred (range 1.37–12.11 min, mean 4.24 min). As we had previously noted that PD1-positive T cells moved quickly in both MCL and NLPHL, it was particularly surprising that these lymphomas differed in the duration of cellular interactions of PD1-positive T cells. We therefore also analyzed the features of the tracks of PD1-positive T cells and compared track length (total length within a track) as well as the displacement (distance between first and last position of a cell). PD1-positive T cells in MCL showed the strongest displacement, with a mean of 12.3 µm (Figure 5, Suppl. Movie 5). In contrast, PD1-positive T cells in NLPHL accumulated a long track that was closely attached to the neoplastic cells with little displacement (Figure 5, Suppl. Movie 6).

## 4. Discussion

This study is, to our knowledge, the first that allows learning about the dynamic presentation of primary human malignant lymphomas. In a proof of principle, we analyzed 20 primary lymph nodes, including 15 cases of malignant lymphomas and five cases of lymphadenitis. In a previous study, we have demonstrated the feasibility and reproducibility of live cell imaging of primary human lymphatic tissue kept alive during the recording period [25]. Now, we have transferred this technique to primary tissues of malignant lymphomas. We generally confirmed that T cells in human lymph nodes move faster than B cells, as previously observed in mice using intravital microscopy. T cells in mice were slightly faster (5–10.8 µm/min) [26,27] when compared with our study (6.68 µm/min for PD1-positive T cells and 5.02 µm/min for CD3-positive T cells). For PD1-positive T cells, which mainly correspond to T_FH_ cells and frequently interact with antigen-presenting cells, we would thus expect a lower overall velocity. The velocity of PD1-positive T cells in malignant lymphomas in this study was comparable with PD1-positive T cells in adenoid tissue in a previous study from our lab (5.8 µm/min) [25]. Most PD1-positive T cells in the present study probably were T_FH_ cells. However, since we only stained for PD1 and not for other T_FH_ cell markers, we cannot exclude that PD1-positive T cells in our study may also include other T cell subsets that can express PD1. Particularly in lymphomas with a non-follicular microenvironment, such as MCL, they may represent activated interfollicular T cells with PD1 expression.

The mean velocity of human B cells in lymphadenitis in our study was 5.74 µm/min, comparable to that of murine B cells (6.4 µm/min) [26]. Whereas the velocity of B cells in lymphadenitis was heterogeneous, the velocity of the B cells in malignant lymphomas was slower and relatively uniform, independent of whether they belonged to the tumor clone. In malignant lymphomas, mutations can influence the correct assembly of the cytoskeleton and thus the ability to migrate (e.g., *ROCK1* mutation in Hodgkin lymphoma, *RHOA* mutations in AITL and DLBCL, and *ACTB* mutations in DLBCL) [3,28,29]. However, since reactive B cells in malignant lymphomas also showed decreased motility, this may rather result from the remodeling of the lymph node architecture and the disturbed function of the immune system. Surprisingly, PD1-positive T cells showed varying velocities in different lymphoma types and did not generally show reduced motility when compared with reactive lymphoid tissue. Lymphomas with a clear germinal center derivation, such as FL, showed relatively slow-moving PD1-positive T cells, whereas the PD1-positive T cells in MCL and NLPHL, which also contain many mantle zone B cells, were relatively fast. Therefore, the velocity of PD1-positive T cells may on one side reflect the niche from which a malignant lymphoma derives. Experiments mostly conducted in mouse models have uncovered the importance of guidance signals including chemokines, integrin ligands and the mechanical cues in controlling the motility of T lymphocytes [30]. The other important determinant influencing the velocity of PD1-positive T cells is the presence and duration of cell–cell contacts. In most lymphomas with fast PD1-positive T cells, only short-term cell–cell contacts occurred between PD1-positive T cells and B cells or CD30-positive cells (such as in MCL, cHL, or AITL). In contrast, in lymphomas with slower PD1-positive T cells (such as MGZL or DLBCL), longer-lasting cellular interactions between PD1-positive T cells and lymphoma cells were usually seen, suggesting the presence of static immunological synapses [31]. Surprisingly, NLPHL, which had fast PD1-positive T cells, was one of the lymphomas with the longest-lasting cell–cell interactions. This is in line with the fact that lymphoma cells interact with rosetting PD1-positive T cells in immunological synapses [21] and suggests, with respect to the lively motility observed in the PD1-positive rosetting T cells, a dynamic immunological synapse as previously described by Friedl et al. between T cells and dendritic cells [31] (also named a kinapse) [32] In contrast, MCL has strong activation of the cyclin D1 oncogene resulting from cyclin D1 translocation into one of the immunoglobulin gene loci, which makes it independent from the stimulation by T_FH_ cells [33]. Likewise, AITL showed relatively fast PD1-positive T cells, with only short-lasting contacts. This may be explained by the frequently observed internalization of the T cell receptor [34] and mutations in downstream effectors of the T-cell receptor signaling pathway [29,35], which help the tumor clone to become independent from external stimuli. Conversely, engagement of the T-cell receptor could result in decreased T cell motility [27].

## 5. Conclusions

In summary, we have for the first time shed light on the motile behavior of primary human malignant lymphomas and their bystander cells. Differences between lymphoma types were observed that complement existing clinical and molecular data and open perspectives of so far unknown dimensions in lymphoma research. Further studies with large case series including translational clinico-pathological aspects are warranted to allow better depicting of the migratory behavior and correlating this with clinical behavior and outcomes.

## Figures and Tables

**Table 1:** Number of cells analyzed in the different cases.

	CD20			PD1			CD3			CD19			CD30		
Lymph Node Type	Number of Movies	From Number of Cases	Number of Cells Analyzed in Total	Number of Movies	From Number of Cases	Number of Cells Analyzed in Total	Number of Movies	From Number of Cases	Number of Cells Analyzed in Total	Number of Movies	From Number of Cases	Number of Cells Analyzed in Total	Number of Movies	From Number of Cases	Number of Cells Analyzed in Total
Lymphadenitis	12	4	1609	16	5	3760	14	5	6337	11	4	2768	2	1	78
AITL	6	1	1689	7	1	5113	-	-	-	-	-	-	5	1	1289
DLBCL	4	1	665	11	2	2855	8	2	3845	8	2	2340	3	1	264
MGZL	3	2	1068	12	2	2282	7	2	2090	-	-	-	12	2	1557
cHL	20	3	3438	20	3	11296	3	2	799	2	1	420	23	3	1661
NLPHL	7	2	1029	9	2	11486	4	2	1837	-	-	-	-	-	-
FL	-	-	-	3	2	1409	-	-	-	4	1	1794	-	-	-
MCL	3	1	583	5	1	4416	2	1	446	-	-	-	2	1	177

## Data Availability

The data presented in this study are available on request from the corresponding author. The raw data (mostly movies) are not publicly available due to large file sizes (several hundred Megabytes for each movie).

## Supplementary Materials

The following are available online at https://www.mdpi.com/article/10.3390/cancers13205208/s1, Suppl. Figure S1. Velocity of CD30-positive reactive cells in lymphadenitis and AITL compared with CD30-positive lymphoma cells in DLBCL, MGZL and cHL (* *p* < 0.05, Kruskal–Wallis test with Dunn’s post-test for multiple comparisons). Each dot represents the mean from one movie. Lymphadenitis, AITL, DLBCL and MCL: one case each, MGZL: two cases, cHL: three cases. Suppl. Figure S2. Number of cell–cell contacts per movie between tumor cells (CD19 or CD30 as indicated) and CD3-positive T cells. Each dot represents the mean from one movie. MGZL and cHL: two cases each; Lymphadenitis and DLBCL: one case each. Suppl. Movie 1. Live cell movie of a mantle cell lymphoma. PD1-positive T cells in red, CD20-positive B cells in blue. 63x magnification. First, live images are shown and later time-stamp routes are drawn according to the reconstructed tracks of the cells (red: PD1; blue: CD20). Suppl. Movie 2. Live cell movie of a nodular lymphocyte predominant Hodgkin lymphoma. PD1-positive T cells in red, CD20-positive B cells in blue. 63x magnification. First, live images are shown and later time-stamp routes are drawn according to the reconstructed tracks of the cells (red: PD1; blue: CD20). Suppl. Movie 3. Live cell movie of a PD1-positive cell (red) and B cell (blue) in a lymphadenitis undergoing a short lasting cell–cell contact. 63x magnification. Suppl. Movie 4. Live cell movie of a PD1-positive cell (red) and B cell (blue) in a lymphadenitis undergoing a long lasting cell–cell contact. 63x magnification. Suppl. Movie 5. Live cell movie of a PD1-positive T cell of a mantle cell lymphoma in red with straight move. Lymphoma cells in blue. 63x magnification. First, live images are shown, then a reconstructed track of the PD1-positive cell is displayed in yellow. Suppl. Movie 6. Live cell movie of a PD1-positive T cell (red) of a nodular lymphocyte predominant Hodgkin lymphoma. Lymphoma (LP) cells in blue. 63x magnification. First, live images are shown, then a reconstructed track of the PD1-positive cell is displayed in yellow.

## References

[1] Swerdlow S.H., Campo E., Harris N.L., Jaffee E.S., Pileri S.A., Stein H., Thiele J., Arber D.A., Hasserjian R.P., le Beau M.M. (2017). WHO Classification of Tumours of Haematopoietic and Lymphoid Tissues.

[2] Swerdlow S.H., Campo E., Pileri S.A., Harris N.L., Stein H., Siebert R., Advani R., Ghielmini M., Salles G.A., Zelenetz A.D. (2016). The 2016 revision of the World Health Organization classification of lymphoid neoplasms. Blood.

[3] Chapuy B., Stewart C., Dunford A.J., Kim J., Kamburov A., Redd R.A., Lawrence M.S., Roemer M.G.M., Li A.J., Ziepert M. (2018). Molecular subtypes of diffuse large B cell lymphoma are associated with distinct pathogenic mechanisms and outcomes. Nat. Med..

[4] Wright G.W., Huang D.W., Phelan J.D., Coulibaly Z.A., Roulland S., Young R.M., Wang J.Q., Schmitz R., Morin R., Tang J. (2020). A Probabilistic Classification Tool for Genetic Subtypes of Diffuse Large B Cell Lymphoma with Therapeutic Implications. Cancer Cell.

[5] Iqbal J., Wright G., Wang C., Rosenwald A., Gascoyne R.D., Weisenburger D.D., Greiner T.C., Smith L., Guo S., Wilcox R.A. (2014). Gene expression signatures delineate biological and prognostic subgroups in peripheral T-cell lymphoma. Blood.

[6] Pastore A., Jurinovic V., Kridel R., Hoster E., Staiger A.M., Szczepanowski M., Pott C., Kopp N., Murakami M., Horn H. (2015). Integration of gene mutations in risk prognostication for patients receiving first-line immunochemotherapy for follicular lymphoma: A retrospective analysis of a prospective clinical trial and validation in a population-based registry. Lancet Oncol..

[7] Lammermann T., Sixt M. (2009). Mechanical modes of ‘amoeboid’ cell migration. Curr. Opin. Cell Biol..

[8] Karolak A., Markov D.A., McCAWLEY L.J., Rejniak K.A. (2018). Towards personalized computational oncology: From spatial models of tumour spheroids, to organoids, to tissues. J. R. Soc. Interface.

[9] Krummel M.F., Bartumeus F., Gérard A. (2016). T cell migration, search strategies and mechanisms. Nat. Rev. Immunol..

[10] Redondo-Muñoz J., García-Pardo A., Teixidó J. (2019). Molecular Players in Hematologic Tumor Cell Trafficking. Front. Immunol..

[11] Body S., Arenys A.E., Miloudi H., Recasens-Zorzo C., Tchakarska G., Moros A., Bustany S., Vidal-Crespo A., Rodriguez V., Lavigne R. (2017). Cytoplasmic cyclin D1 controls the migration and invasiveness of mantle lymphoma cells. Sci. Rep..

[12] Corcione A., Arduino N., Ferretti E., Raffaghello L., Roncella S., Rossi D., Fedeli F., Ottonello L.C., Trentin L., Dallegri F. (2004). CCL19 and CXCL12 Trigger in Vitro Chemotaxis of Human Mantle Cell Lymphoma B Cells. Clin. Cancer Res..

[13] Ding N., Ping L., Feng L., Zheng X., Song Y., Zhu J. (2014). Histone deacetylase 6 activity is critical for the metastasis of Burkitt’s lymphoma cells. Cancer Cell Int..

[14] Lu X., Chen J., Malumbres R., Gil E.C., Helfman D.M., Lossos I.S. (2007). HGAL, a lymphoma prognostic biomarker, interacts with the cytoskeleton and mediates the effects of IL-6 on cell migration. Blood.

[15] Linke F., Zaunig S., Nietert M., Von Bonin F., Lutz S., Dullin C., Janovská P., Beissbarth T., Alves F., Klapper W. (2016). WNT5A: A motility-promoting factor in Hodgkin lymphoma. Oncogene.

[16] Van Loo P., Tousseyn T., Vanhentenrijk V., Dierickx D., Malecka A., Bempt I.V., Verhoef G., Delabie J., Marynen P., Matthys P. (2009). T-cell/histiocyte-rich large B-cell lymphoma shows transcriptional features suggestive of a tolerogenic host immune response. Haematologica.

[17] Calabretta E., D’Amore F., Carlo-Stella C. (2019). Immune and Inflammatory Cells of the Tumor Microenvironment Represent Novel Therapeutic Targets in Classical Hodgkin Lymphoma. Int. J. Mol. Sci..

[18] Lenz G., Wright G., Dave S., Xiao W., Powell J., Zhao H., Xu W., Tan B., Goldschmidt N., Iqbal J. (2008). Stromal Gene Signatures in Large-B-Cell Lymphomas. N. Engl. J. Med..

[19] Kumar D., Xu M.L. (2018). Microenvironment Cell Contribution to Lymphoma Immunity. Front. Oncol..

[20] Townsend W., Pasikowska M., Yallop D., Phillips E., Patten P., Salisbury J., Marcus R., Pepper A., Devereux S. (2019). The architecture of neoplastic follicles in follicular lymphoma; analysis of the relationship between the tumor and follicular helper T cells. Haematologica.

[21] Bein J., Thurner L., Hansmann M., Hartmann S. (2020). Lymphocyte predominant cells of nodular lymphocyte predominant Hodgkin lymphoma interact with rosetting T cells in an immunological synapse. Am. J. Hematol..

[22] Laurent C., Fazilleau N., Brousset P. (2010). A novel subset of T-helper cells: Follicular T-helper cells and their markers. Haematologica.

[23] Qin L., Waseem T., Sahoo A., Bieerkehazhi S., Zhou H., Galkina E.V., Nurieva R. (2018). Insights Into the Molecular Mechanisms of T Follicular Helper-Mediated Immunity and Pathology. Front. Immunol..

[24] Donnadieu E., Michel Y., Hansmann M.-L. (2019). Live Imaging of Resident T-Cell Migration in Human Lymphoid Tissue Slices Using Confocal Microscopy. T-Cell Motility.

[25] Donnadieu E., Reisinger K.B., Scharf S., Michel Y., Bein J., Hansen S., Loth A.G., Flinner N., Hartmann S., Hansmann M.-L. (2020). Landscape of T Follicular Helper Cell Dynamics in Human Germinal Centers. J. Immunol..

[26] Miller M.J., Wei S.H., Parker I., Cahalan M.D. (2002). Two-Photon Imaging of Lymphocyte Motility and Antigen Response in Intact Lymph Node. Science.

[27] Stoll S., Delon J., Brotz T., Germain R.N. (2002). Dynamic Imaging of T Cell-Dendritic Cell Interactions in Lymph Nodes. Science.

[28] Liu Y., Razak F.R.A., Terpstra M., Chan F.C., Saber A., Nijland M., Van Imhoff G., Visser L., Gascoyne R., Steidl C. (2014). The mutational landscape of Hodgkin lymphoma cell lines determined by whole-exome sequencing. Leukemia.

[29] Vallois D., Dobay M.P.D., Morin R.D., Lemonnier F., Missiaglia E., Juilland M., Iwaszkiewicz J., Fataccioli V., Bisig B., Roberti A. (2016). Activating mutations in genes related to TCR signaling in angioimmunoblastic and other follicular helper T-cell–derived lymphomas. Blood.

[30] Fowell D.J., Kim M. (2021). The spatio-temporal control of effector T cell migration. Nat. Rev. Immunol..

[31] Friedl P., Bröcker E.-B. (2002). TCR triggering on the move: Diversity of T-cell interactions with antigen-presenting cells. Immunol. Rev..

[32] Dustin M.L. (2008). Visualization of Cell-Cell Interaction Contacts-Synapses and Kinapses. Multichain Immune Recognit. Recept. Signal..

[33] Jares P., Colomer D., Campo E. (2007). Genetic and molecular pathogenesis of mantle cell lymphoma: Perspectives for new targeted therapeutics. Nat. Rev. Cancer.

[34] Alikhan M., Song J.Y., Sohani A.R., Moroch J., Plonquet A., Duffield A.S., Borowitz M.J., Jiang L., Bueso-Ramos C., Inamdar K. (2016). Peripheral T-cell lymphomas of follicular helper T-cell type frequently display an aberrant CD3−/dimCD4+ population by flow cytometry: An important clue to the diagnosis of a Hodgkin lymphoma mimic. Mod. Pathol..

[35] Heavican T.B., Bouska A., Yu J., Lone W., Amador C., Gong Q., Zhang W., Li Y., Dave B.J., Nairismägi M.-L. (2019). Genetic drivers of oncogenic pathways in molecular subgroups of peripheral T-cell lymphoma. Blood.

