# Supplementary figures and images for: Landscape of 4D Cell Interaction in Hodgkin and Non-Hodgkin Lymphomas
Source: Cancers (Basel). 2021 Oct 17;13(20):5208. doi: 10.3390/cancers13205208 (PMC8534096; doi:10.3390/cancers13205208)

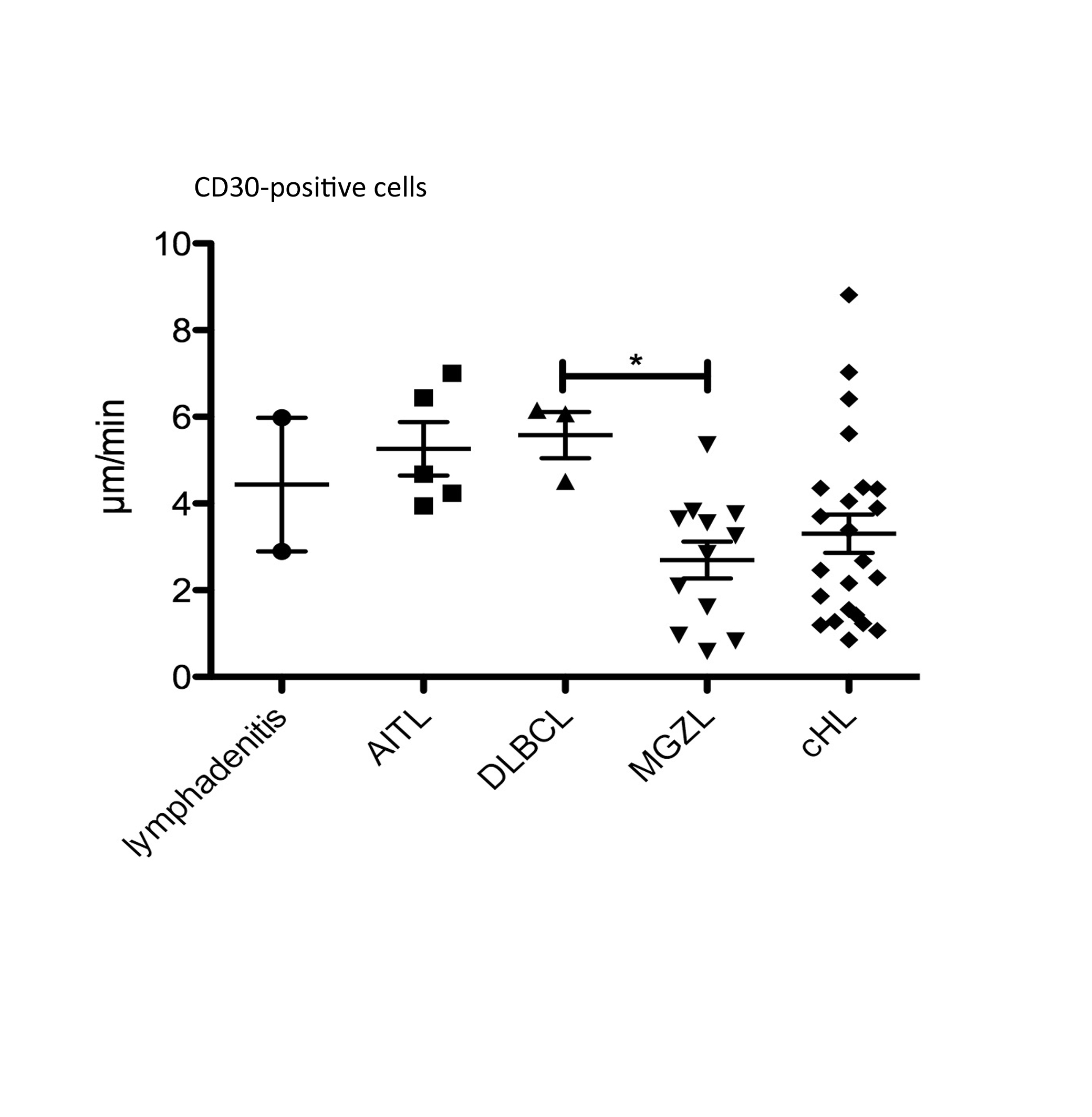

Supplement: Supplementary file 1 [file cancers-13-05208-s001.zip › Suppl. Data 4D Landscapes final/Suppl. Figure 1.jpg]

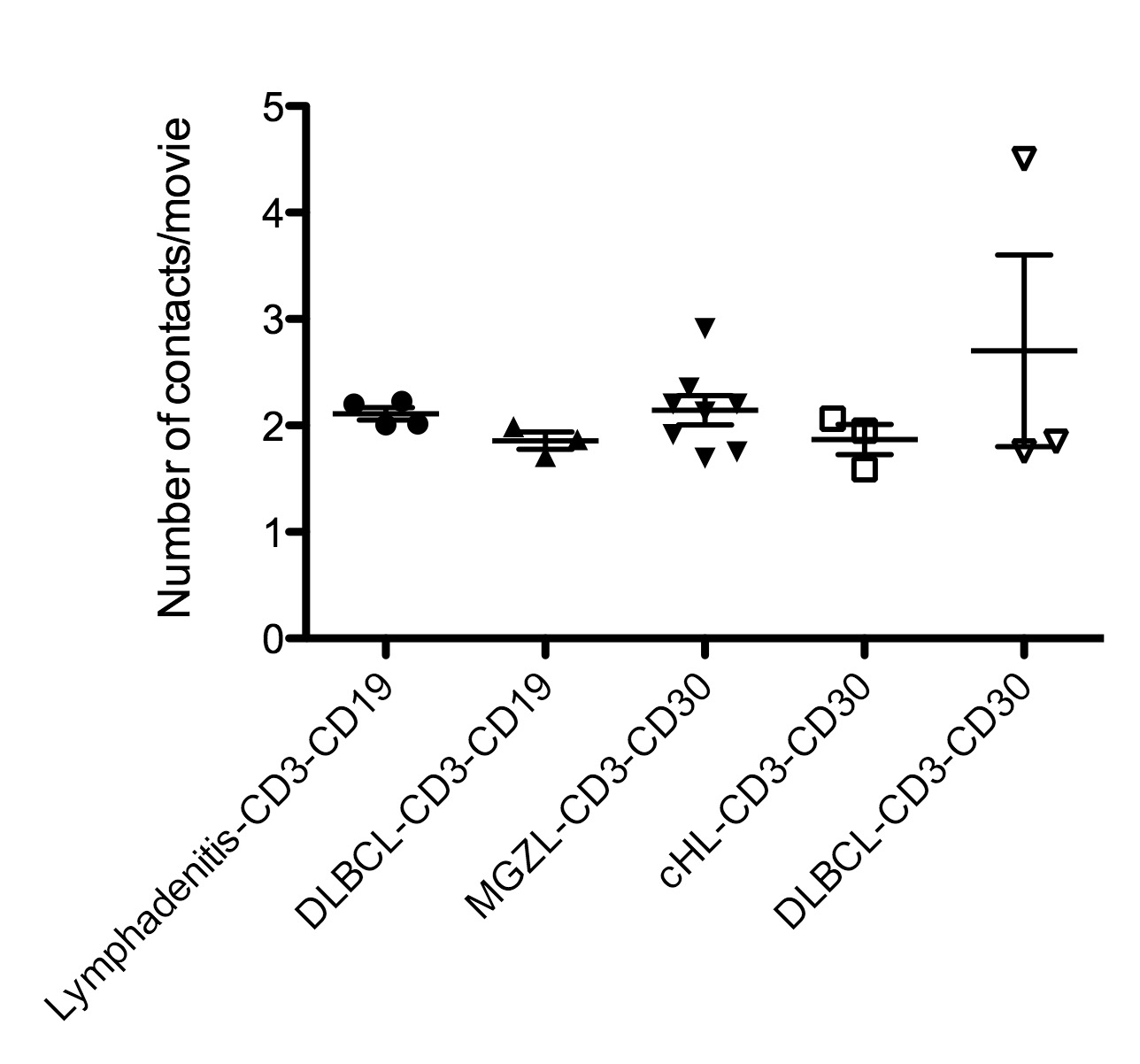

Supplement: Supplementary file 1 [file cancers-13-05208-s001.zip › Suppl. Data 4D Landscapes final/Suppl. Figure 2.jpg]
